# Thickness-Dependent DC Electrical Breakdown of Polyimide Modulated by Charge Transport and Molecular Displacement

**DOI:** 10.3390/polym10091012

**Published:** 2018-09-11

**Authors:** Daomin Min, Yuwei Li, Chenyu Yan, Dongri Xie, Shengtao Li, Qingzhou Wu, Zhaoliang Xing

**Affiliations:** 1 State Key Laboratory of Electrical Insulation and Power Equipment, Xi’an Jiaotong University, Xi’an 710049, China; mirana111@stu.xjtu.edu.cn (Y.L.); leo-chenyu.yan@stu.xjtu.edu.cn (C.Y.); xiedongri@stu.xjtu.edu.cn (D.X.); 2 Institute of Fluid Physics, China Academy of Engineering Physics, Mianyang 621900, China; wuqingzhou@163.com; 3 State Key Laboratory of Advanced Power Transmission Technology, Beijing 102209, China; zhaoweimei@sgri.sgcc.com.cn

**Keywords:** charge transport, DC electrical breakdown, free volume, molecular chain displacement, sample thickness

## Abstract

Polyimide has excellent electrical, thermal, and mechanical properties and is widely used as a dielectric material in electrical equipment and electronic devices. However, the influencing mechanism of sample thickness on electrical breakdown of polyimide has not been very clear until now. The direct current (DC) electrical breakdown properties of polyimide as a function of thickness were investigated by experiments and simulations of space charge modulated electrical breakdown (SCEB) model and charge transport and molecular displacement modulated (CTMD) model. The experimental results show that the electrical breakdown field decreases with an increase in the sample thickness in the form of an inverse power function, and the inverse power index is 0.324. Trap properties and carrier mobility were also measured for the simulations. Both the simulation results obtained by the SCEB model and the CTMD model have the inverse power forms of breakdown field as a function of thickness with the power indexes of 0.030 and 0.339. The outputs of the CTMD model were closer to the experiments. This indicates that the displacement of a molecular chain with occupied deep traps enlarging the free volume might be a main factor causing the DC electrical breakdown field of polyimide varying with sample thickness.

## 1. Introduction

Polyimide has excellent thermal stability, outstanding electrical properties, and high mechanical strength and is widely used as a dielectric material in power capacitors for energy storage [1,2,3], the solar array of satellite [4,5], and electronic devices [6], and so on. As the voltage level increases and the devices are required to be further miniaturized, dielectric materials with high electrical strength need to be developed. Insulation failures of solid dielectrics under electrical stresses can cause permanent damage to the materials and shorten the service life of electrical equipment or electronic devices. Therefore, the electrical breakdown mechanism of solid dielectrics is a research focus in the fields of dielectric energy storage and electrical insulation.

The electrical breakdown field of dielectric materials depends on sample thickness. The electrical breakdown field of polyimide film under direct current (DC) voltage was measured at various sample thicknesses and various temperatures. The DC electrical breakdown field decreases with an increase in the sample thickness [7]. The relation between the DC electrical breakdown field and sample thickness obeys an inverse power law with power indexes of 0.16 and 0.25 at 300 °C and 400 °C, respectively. Regardless of the thickness of the film, the DC breakdown field decreases as the temperature increases in a range from 25 to 400 °C [7]. Similar inverse power law dependence of the DC electrical breakdown field on sample thickness was also observed on polytetrafluoroethylene [8] and an acrylic dielectric elastomer [9]. In addition, electrical breakdown properties of polyimide, polyethylene terephthalate, polyetheretherketone, and polyethersulfone were studied under alternating current (AC) voltage with a rising rate of 500 Vs^−1^, and it was found that the AC electrical breakdown field decreases with an increase in the sample thickness in an inverse power-law form for all the four polymers [10]. The electrical breakdown field under nanosecond pulses was also found to be a decreasing function of sample thickness on polyethylene, polytetrafluoroethylene, polymethyl methacrylate, and nylon [11].

There are several breakdown mechanisms such as electron avalanche breakdown [12,13], electromechanical breakdown [12,13], free volume breakdown, and space charge modulated electrical breakdown [14,15,16], to interpret the sample thickness effect on the electrical breakdown of polymers. In the electron avalanche breakdown [12,13], free electrons in the conduction band of a dielectric material could move a certain distance under the electric field, resulting in an energy gain of electrons. When the energy exceeds the band gap energy, electrons in the valence band might be excited to the conduction band, causing chemical bond scission. The released electrons further collided with and ionized other matrix atoms to cause an electronic avalanche effect, resulting in the multiplication of local current and eventually triggering electrical breakdown. Since a critical number of generations of electrons are required to be produced over the sample thickness by impact ionization, the electrical breakdown field decreases with an increase in the sample thickness [12,13]. The Stark–Garton model of electromechanical breakdown has been widely used to predict the breakdown strength of thermoplastics, and the electromechanical breakdown strength depends on Young’s modulus and dielectric constant for temperature-sensitive polymers [12,13]. Since both the electrostatic compressive stress generated by the electrostatic attraction of two electrodes and the opposing elastic stress depend on sample thickness, the electrical breakdown field is a decreasing function of sample thickness [12,13]. Moreover, in the free volume breakdown theory, it was assumed that the electrical breakdown field of a polymer depends on the longest average free path of electrons. Electrons are accelerated in free volume, and their average free path depended on the maximum length of free volume. When the electron obtained enough energy in free volume to overcome the potential barrier, the local current was multiplied to heat the material to an extremely high temperature, and eventually resulting in electrical breakdown [17]. The longest free path is a function of sample size from a statistical point of view, so the electrical breakdown strength is related to the sample thickness. However, the relations between DC electrical breakdown strength and sample thickness obtained by the models of electron avalanche breakdown, electromechanical breakdown, and free volume breakdown do not satisfy the inverse power law as observed in experiments [12,17].

It has demonstrated that charges are injected into and space charges are accumulated inside a dielectric material under the application of a high electric field [18,19,20,21]. The electric field inside the dielectric material will be distorted by the accumulated space charges, and electrical breakdown occurs when the maximum local electric field exceeds a threshold value [20,22]. Then, a model considering the formation of space charges and the distortion of electric field was established to simulate the DC electrical breakdown properties of low-density polyethylene, and it was found that the breakdown strength is an inverse power function of sample thickness [14]. A similar model was used to investigate the electrical breakdown properties of oil-impregnated paper [15] and low-density polyethylene-based nanocomposites [16]. The simulation results are in good agreement with experiments [15,16]. These results show that the electric field distortion caused by the space charge accumulation under a high field may play an important role in the breakdown characteristics of polyimide. In addition, the macroscopic dielectric properties of polymers are under the influence of molecular chain motion [23]. A molecular chain with a deep trapped charge can move toward the electrode via the Coulomb force under the high electric field, resulting in the elongation of free volume [24,25,26]. The elongation of free volume caused by the displacement of the molecular chain associating with the accumulation of space charges and the distortion of electric field may determine the electrical breakdown behavior of polymeric materials. Therefore, in the present paper, two electrical breakdown models based on the space charge effect and molecular chain displacement dynamics are utilized to investigate sample thickness dependent the DC electrical breakdown properties of polyimide.

## 2. Experimental Procedure

Polyimide films (type: 6051) with various thicknesses were purchased from Baoying County Seiko Insulation Materials Co., Ltd., Yangzhou, China. Firstly, polyamide acid was synthesized by polycondensation between phthalic acid dianhydride and diamino diphenyl ether in a high polar solution. Films were then prepared by the solution of polyamide acid through casting and stretching methods. Then, polyimide films were obtained via drying and imidization of polyamide acid at high temperature. The thickness of polyimide films ranges from 25 to 250 μm. Before the experiments, all the samples were heated in a chamber at about 100 °C for 12 h to eliminate the humidity inside. Au electrodes with a radius of 15 mm were sputtered by ion sputter coater on both sides of the sample. Then, a Broadband Dielectric Spectrometer (Concept 80, Novocontrol Technologies, Frankfurt, Germany) was used to measure the complex permittivity of polyimide with a thickness of 100 μm at room temperature. The voltage applied on the sample was 1 V_rms_ and the frequency was from 10^−2^ to 10^5^ Hz.

A thermal stimulation depolarization current (TSDC, Concept 90, Novocontrol technologies, Frankfurt, Germany) was carried out on a polyimide sample with a thickness of 100 μm to investigate its trap distribution characteristics. The sample was placed in the vacuum chamber of TSDC device. The sample was first heated to 180 °C and then polarized for 30 min at an applied voltage of 250 V. After the polarization, the sample was cooled to −100 °C by liquid nitrogen and then short-circuited for 10 min. Thereafter, the sample was heated to 290 °C with a heating rate of 3 °Cmin^−1^. At the same time, the depolarization current was measured using a pico-ammeter (Keithley 6517B, Beaverton, OR, USA).

Surface potential decay (SPD) measurement was performed on polyimide films with a thickness of 25 μm to investigate potential decay and carriers mobility characteristics. The SPD measurement includes two processes, which are the charging process and detection process. When a voltage is applied to the needle electrode, energetic charges emitted from the needle electrode will impact with air molecules and cause ionization, which is called corona discharging. During the corona discharging, a large amount of positive and negative charges are generated. Driven by the electric field of the grid electrode, charges will drift toward and be deposited on the sample surface. In this paper, voltages applied to the needle and gate electrodes were 12 and 8 kV, respectively, for positive corona discharging, and were −12 and −8 kV respectively for negative corona discharging. The charging time on polyimide sample was 2 min. After the charging process, the surface potential of samples was measured by a high-voltage electrostatic voltmeter (P0865, Trek, Lockport, NY, USA) with a non-contact probe (3453ST, Trek, Lockport, NY, USA).

The DC electrical breakdown fields of polyimide films with different thicknesses from 25 to 250 μm were measured using a computer-controlled voltage breakdown test device (HJC-100kV, Huayang Instrument Co., Ltd., Yangzhong, China). The DC electrical breakdown experiments were carried out at 30 °C using spherical copper electrodes with a diameter of 25 mm in transformer oil (Kramai25#, China National Petroleum Corporation, Karamay, China). A DC voltage with a ramping rate of 1 kVs^−1^ was applied to the samples until the breakdown occurring. The breakdown voltages were recorded by a computer. The breakdown tests were repeated at least 15 times for each thickness of the samples. The average of all the data was taken as the electrical breakdown field for the sample with corresponding thickness.

## 3. Experimental Results

**Figure 1:**
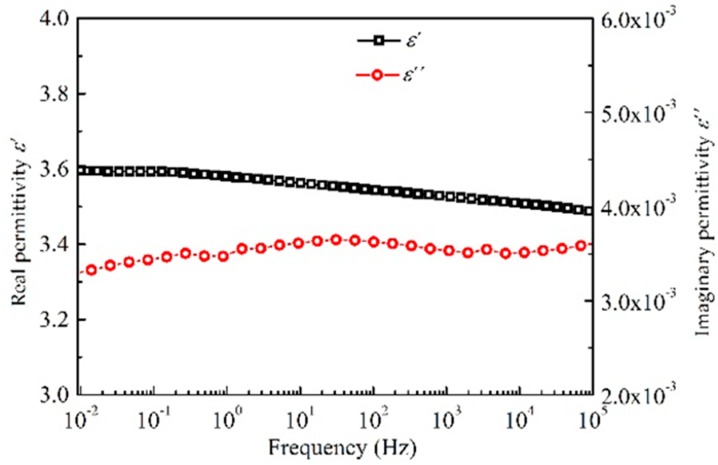
The real and imaginary parts of relative complex permittivity of polyimide as a function of frequency at room temperature.

Figure 1 shows the real and imaginary parts of relative complex permittivity, *ε*’ and *ε*”, as a function of frequency in semi-logarithmic coordinates obtained from the polyimide sample at room temperature. It can be seen that the real part of the relative complex permittivity increases slightly from 3.5 to 3.6 with a decrease in frequency. The imaginary part of the relative complex permittivity is lower than 3.6 × 10^−3^ in the frequency range from 10^−2^ to 10^5^ Hz. The dielectric relaxation strength of the relaxation peak around 30 Hz is very small, which means the dipolar moment is very low. The low dielectric loss *ε*”/*ε*’ indicates that Joule heating generated by the orientation of dipoles during the DC electrical breakdown experiments at room temperature can be neglected.

**Figure 2:**
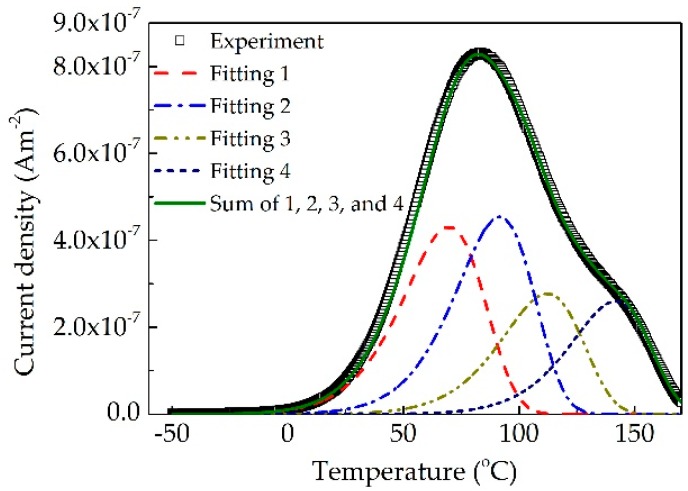
Thermal stimulation depolarization current (TSDC) experimental and fitting results of polyimide. Symbols and solid curves represent experimental and fitting results, respectively. The fitting curves are calculated by Equation (1).

Figure 2 demonstrates the TSDC experimental results of polyimide. Thermally stimulated relaxation processes can be observed at the temperature range from 10 to 170 °C. One obvious relaxation peak is around 69 °C, while another relaxation peak may locate around 135 °C. There may be other relaxation processes between 69 and 135 °C. In order to reveal the thermally stimulated processes and their activation energies, the experimental results in Figure 2 were analyzed by the classical TSDC theory [27]:

(1) $$j_{\mathrm{TSC}}\left(T\right)=B\exp\left[-\frac{E_{A}}{k_{B}T}-\frac{1}{\beta \tau_{0}}\int_{T_{0}}^{T^{\prime }}\exp\left(-\frac{E_{A}}{k_{B}T}\right)dT\right]$$

where *j*_TSC_(*T*) is the thermally stimulated depolarization current density in Am^−2^, *B* is a constant in Am^−2^, *E*_A_ is the activation energy of the relaxation process in eV, *τ*_0_ is the relaxation time constant in s, *β* is the heating rate in °Cs^−1^, *k*_B_ is the Boltzmann constant, *T*_0_ is the initial temperature of the sample at the beginning of the heating process in °C, and *T* is the temperature of the sample after heating in °C.

The TSDC experimental results were fitted by Equation (1), and four relaxation peak components could be obtained. As shown in Figure 2, it can be seen that the fitting results are in good agreement with the experiments. We can determine the peak temperature, activation energy and relaxation time for the four relaxation processes listed in Table 1. The activation energies of four peaks at 69, 87, 109 and 135.5 °C are 0.60, 0.65, 0.70 and 0.83 eV, respectively. As the temperature at the relaxation peak increase, the corresponding activation energy increases. The three peaks at 69, 87 and 109 °C may correspond to shallow traps that assist carriers hopping process in polyimide, while the peak at 135.5 °C may correspond to deep traps that can capture mobile carriers and accumulate space charges. The energy of deep traps is consistent with the results obtained from the Arrhenius relation between conductivity and temperature [28]. The activation energy 0.83 eV of relaxation peak at 135.5 °C was used as deep trap energy parameter in the simulation of electrical breakdown.

**Figure 3:**
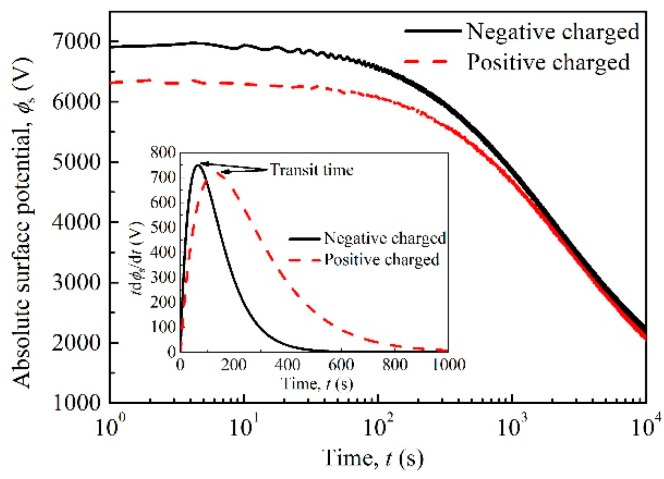
Surface potentials of polyimide charged by negative corona discharging and positive corona discharging as a function of time at room temperature.

Figure 3 shows the surface potential experimental results of samples charged by negative corona discharging and positive corona discharging as a function of time. During the charging process, negative charges and positive charges are deposited on the surface of polyimide and electric fields are built-up inside the material. After the charging, surface charges inject into and move toward the grounded electrode in the bulk of material. The migration of charges leads to the decay of surface potential. The decay rate of surface potential varies before and after the injected charge carriers flow out of the dielectric material [29,30], as shown in Figure 3. The time when the front charge carriers arrives at the grounded electrode is defined as transit time *t*_T_. The transit point existing at the beginning of the potential decay curve can represent the mobility of carriers controlled by shallow traps [29,30]. In order to obtain the transit time *t*_T_, the potential decay results were fitted by an exponential function [31], and then we obtained the relation between *t*d*φ*_s_/d*t* and *t*. The time corresponding to the peaks can be regarded as transit time *t*_T_, and is used to calculate carrier mobility controlled by shallow traps according to the following equation [29],

(2) $$\mu_{0\left(e,h\right)}=d^{2}/\phi_{s0}t_{T}$$

Here *μ*_0_ is carrier mobility controlled by shallow traps in m^2^V^−1^s^−1^, *d* is the thickness of sample in m, *φ*_s0_ represents the initial surface potential in V. The subscripts have the following meaning: (e) for electrons and (h) for holes. By calculation, the hole and electron mobilities controlled by shallow traps are 1.80 × 10^−14^ m^2^V^−1^s^−1^ and 3.67 × 10^−14^ m^2^V^−1^s^−1^ respectively.

**Figure 4:**
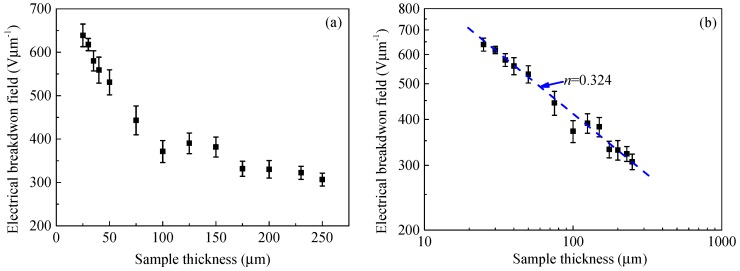
Experimental results of DC electrical breakdown field of polyimide at various thicknesses in linear coordinates (**a**) and in double logarithmic coordinates (**b**).

Figure 4 shows the experimental results of the DC electrical breakdown field of the polyimide film, *F*_b_, as a function of thickness, *d*, at room temperature. As shown in Figure 4a, the DC electrical breakdown field of polyimide films decreases with an increase in sample thickness. For example, the electrical breakdown field is 639 Vμm^−1^ at a sample thickness of 25 μm, 371 Vμm^−1^ at 100 μm, and 307 Vμm^−1^ at 250 μm. In addition, the derivative of DC electrical breakdown field with respect to sample thickness d*F*_b_/d*d* decreases with the increase in sample thickness. The relation between the DC electrical breakdown field and sample thickness looks like an inverse power function. Accordingly, we change the linear coordinates in Figure 4a to double logarithmic coordinates in Figure 4b. It can be seen from Figure 4b that the DC electrical breakdown field of polyimide is linear with sample thickness under double logarithmic coordinates. The electrical breakdown field decreases with increasing sample thickness in an inverse power function form, *F*_b_ = *kd*^−*n*^. The power index calculated from the experimental results is 0.324. Similar results were also observed by Diaham et al. [7], and the power index of sample thickness dependent DC electrical breakdown field is in a range of 0.16 to 0.25.

## 4. Numerical Analysis of Sample Thickness-Dependent DC Electrical Breakdown

At a high electric field, charges in an electrode can be injected into a dielectric material via Schottky thermionic emission or quantum mechanical tunneling. The accumulation of space charges under high voltage causes electric field distortion inside the dielectric material [14,15,16,32,33]. The maximum local electric field *F*_max_ would be much higher than the applied electric field. Electrical breakdown would occur when *F*_max_ exceeds a critical value *F*_c_. This is named as the space charge modulated electrical breakdown model (SCEB) or bipolar charge transport model [14,15,16]. In addition, a charge transport and molecular displacement modulated electrical breakdown model (CTMD) is utilized to investigate the sample thickness dependent DC electrical breakdown of polyimide [26]. Since free volume exists in polymers, it may be enlarged by the displacement of molecular chains with trapped charges by the Coulomb force under the high electric field [24,25]. Electrons are accelerated in the free volume under the effect of the electric field to obtain energy. The energy *w* of electrons gained from the electric field depends on the local electric field *F* and the length of the free volume *λ*, namely *w* = *eFλ*. The energy of electrons increases with an increase in the local electric field and the enlargement of free volume caused by the displacement of molecular chains. When the maximum energy of electrons *w*_max_ is higher than the deep traps energy *E*_T_, electrons have enough energy to jump over the potential barrier, which will result in electrical breakdown [26].

### 4.1. Numerical Analysis by Space Charge Modulated Electrical Breakdown (SCEB) Model

#### 4.1.1. SCEB Model

**Figure 5:**
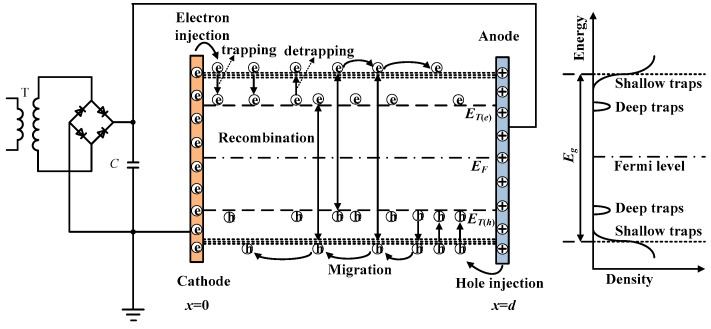
Schematic diagram of space charge modulated electrical breakdown model. Here, *E*_μ(e)_ and *E*_μ(h)_ are the mobility edges of electrons and holes, *E*_T(e)_ and *E*_T(h)_ are the energy of deep traps for electrons and holes, *E*_F_ is the Fermi level, and *E*_g_ is the band gap.

Figure 5 shows the schematic diagram of the SCEB model for a dielectric material under DC voltage [14,15,16]. The cathode and anode are located at *x* = 0 and *x* = *d*, respectively. After a high electric field is applied on the material, charges are injected from metal electrodes into the dielectric material. Electrons and holes on metal electrodes have to overcome effective potential barriers between the electrodes and the dielectric material. After the charges are injected into the dielectric material, they will migrate in the extended states or shallow traps above the mobility edge of electrons *E*_μ(e)_ and below the mobility edge of holes *E*_μ(h)_, which are named as free electrons and free holes. In addition, deep traps confined in a single energy level, *E*_T(e)_ and *E*_T(h)_, are considered in the simulation model for electrons and holes, respectively. When free electrons and holes trapped by deep traps, space charge accumulation is formed gradually. The trapped charges can be released from deep traps by the thermal activation process when they get enough energy. When free electrons and holes encounter each other in the material, charge recombination will occur. The mathematical equations of the SCEB model are given in the following section.

Electrons and holes are assumed to be injected into the dielectric material from cathode and anode, respectively, by Schottky thermionic emission. The current densities of charge injections are determined by effective injection barriers between the sample and its electrodes, electric fields at the interfaces, and temperature [14,15,16,32,33].

(3) $$j_{in(e)}(t)=AT^{2}\exp\left[-\frac{E_{\mathrm{in}(e)}}{k_{B}T}\right]\exp\left[\frac{\sqrt{eF(0,t)/4\pi \varepsilon_{0}\varepsilon_{r}}}{k_{B}T}\right]\;$$

(4) $$j_{\mathrm{in}(h)}(t)=AT^{2}\exp\left[-\frac{E_{\mathrm{in}(h)}}{k_{B}T}\right]\exp\left[\frac{\sqrt{eF(d,t)/4\pi \varepsilon_{0}\varepsilon_{r}}}{k_{B}T}\right]\;$$

Here, *j*_in(e)_ and *j*_in(h)_ represent the current densities caused by electrons injected from the cathode and holes injected from the anode into the dielectric material, respectively, in Am^−2^; *E*_in(e)_ and *E*_in(h)_ are the effective injection barriers for electrons and holes, respectively, in eV; *A* is the Richardson constant (=1.20 × 10^6^ Am^−2^K^−2^); *e* is the elementary charge in C; *ε*_0_ is the vacuum permittivity in Fm^−1^; *ε*_r_ is the relative permittivity of a dielectric material; and *t* is the time after applying a voltage in s. Furthermore, *F(*0, *t*) and *F*(*d*, *t*) are the electric fields at the interfaces of *x* = 0 and *x* = *d*, respectively.

The injected charges migrate in the dielectric material, and the current densities of electrons and holes are expressed as follows [14,15,16,32,33]:

(5) $$j_{c\left(e\right)}(x,t)=q_{\mathrm{free}\left(e\right)}\left(x,t\right)\mu_{0\left(e\right)}F\left(x,t\right)$$

(6) $$j_{c\left(h\right)}(x,t)=q_{\mathrm{free}\left(h\right)}\left(x,t\right)\mu_{0\left(h\right)}F\left(x,t\right)$$

where, *j*_c(e)_ and *j*_c(h)_ are the conduction current densities formed by electrons and holes, respectively, in Am^−2^; *q*_free(e)_ and *q*_free(h)_ are the density of free electrons and free holes, respectively, in Cm^−3^; and *F* is the electric field in Vμm^−1^.

The variations of charges in the dielectric material satisfy the charge continuity equations with the consideration of charge trapping, detrapping, and recombination processes. Traps in this model are assumed to be with single trap energy when charges are trapping and detrapping, and recombination occurs when electrons and holes encounter each other. The following equations demonstrate such a series of processes [14,15,16,32,33]:

(7) $$\begin{array}{l} \frac{\partial q_{\mathrm{free}\left(e\right)}\left(x,t\right)}{\partial t}+\frac{\partial j_{c\left(e\right)}\left(x,t\right)}{\partial t}=\\ -P_{\mathrm{tr}\left(e\right)}q_{\mathrm{free}\left(e\right)}\left(1-\frac{q_{\mathrm{trap}\left(e\right)}}{eN_{T\left(e\right)}}\right)+P_{\mathrm{de}\left(e\right)}q_{\mathrm{trap}\left(e\right)}-R_{e\mu ,h\mu }q_{\mathrm{free}\left(e\right)}q_{\mathrm{free}\left(h\right)}-R_{e\mu ,\mathrm{ht}}q_{\mathrm{free}\left(e\right)}q_{\mathrm{trap}\left(h\right)} \end{array}$$

(8) $$\frac{\partial q_{\mathrm{trap}\left(e\right)}\left(x,t\right)}{\partial t}=P_{\mathrm{tr}\left(e\right)}q_{\mathrm{free}\left(e\right)}\left(1-\frac{q_{\mathrm{trap}\left(e\right)}}{eN_{T\left(e\right)}}\right)-P_{\mathrm{de}\left(e\right)}q_{\mathrm{trap}\left(e\right)}-R_{\mathrm{et},h\mu }q_{\mathrm{trap}\left(e\right)}q_{\mathrm{free}\left(h\right)}\;$$

(9) $$\begin{array}{l} \frac{\partial q_{\mathrm{free}\left(h\right)}\left(x,t\right)}{\partial t}+\frac{\partial j_{c\left(h\right)}\left(x,t\right)}{\partial t}=\\ -P_{\mathrm{tr}\left(h\right)}q_{\mathrm{free}\left(h\right)}\left(1-\frac{q_{\mathrm{trap}\left(h\right)}}{eN_{T\left(h\right)}}\right)+P_{\mathrm{de}\left(h\right)}q_{\mathrm{trap}\left(h\right)}-R_{e\mu ,h\mu }q_{\mathrm{free}\left(e\right)}q_{\mathrm{free}\left(h\right)}-R_{\mathrm{et},h\mu }q_{\mathrm{trap}\left(e\right)}q_{\mathrm{free}\left(h\right)} \end{array}$$

(10) $$\frac{\partial q_{\mathrm{trap}\left(h\right)}\left(x,t\right)}{\partial t}=P_{\mathrm{tr}\left(h\right)}q_{\mathrm{free}\left(h\right)}\left(1-\frac{q_{\mathrm{trap}\left(h\right)}}{eN_{T\left(h\right)}}\right)-P_{\mathrm{de}\left(h\right)}q_{\mathrm{trap}\left(h\right)}-R_{e\mu ,\mathrm{ht}}q_{\mathrm{free}\left(e\right)}q_{\mathrm{trap}\left(h\right)}\;$$

Here, *q*_trap(e)_ and *q*_trap(h)_ are the density of trapped electrons and trapped holes, respectively, in Cm^−3^; while *R*_eµ,hμ_, *R*_et,ht_, and *R*_et,hμ_ are the recombination coefficients in m^3^C^−1^s^−1^; with subscripts having the following meanings: eμ for mobile electrons, et for trapped electrons, hμ for mobile holes, and ht for trapped holes. In addition, *P*_tr_ is the trapping probability in s^−1^; *P*_de_ represents detrapping probability in s^−1^; and *N*_T_ is the density of deep traps in m^−^^3^.

The trapping probability of a mobile charge into a deep trap has a positive correlation with carrier mobility and the density of deep traps, and is inversely proportional to permittivity, namely, *P*_tr(e,h)_ = *eN*_T(e,h)_/*ε*_0_*ε*_r_ [34]. The detrapping probability of a trapped charge at a deep trap center is determined by trap energy and temperature, *P*_de(e,h)_ = *υ*_ATE_exp(−*E*_T(e,h)_/*k*_B_*T*) [14,15,16,32,33]. Here, *υ*_ATE_ represents the attempt-to-escape frequency in s^−1^. Based on the Langevin recombination model, the recombination coefficient between free electrons and free holes can be expressed as, *R*_eμ,hμ_ = (*μ*_0(e)_ + *μ*_0(h)_)/*ε*_0_*ε*_r_ [35]. According to the Shockley–Read–Hall model [36], trap-assisted recombination coefficients between free electrons and trapped holes and that between trapped-electron and free-hole can be expressed as, *R*_eμ,ht_ = *μ*_0(e)_/*ε*_0_*ε*_r_ and *R*_et,hμ_ = *μ*_0(h)_/*ε*_0_*ε*_r_, respectively. The units of recombination coefficients are m^3^C^−1^s^−1^.

After space charges are accumulated in the polymeric material, the electric potential and electric field inside the material are distorted. The relation between electric potential *φ* and space charge is determined by Poisson’s equation [14,15,16,32,33]:

(11) $$\frac{\partial^{2}\phi \left(x,t\right)}{\partial x^{2}}=-\frac{q_{\mathrm{free}\left(e\right)}\left(x,t\right)+q_{\mathrm{trap}\left(e\right)}\left(x,t\right)+q_{\mathrm{free}\left(h\right)}\left(x,t\right)+q_{\mathrm{trap}\left(h\right)}\left(x,t\right)}{\varepsilon_{0}\varepsilon_{r}}\;$$

When we solve Poisson’s equation, a boundary condition should be given. During the DC electrical breakdown experiment, a positive ramping voltage with a rising rate of *k*_ramp_ is applied to the sample until electrical breakdown occurs. Accordingly, the electric potential is set to be zero on the cathode and equals the external applied voltage on the anode. In addition, the electric potential on the anode or the external applied voltage is proportional to time after the application of voltage. The boundary condition is expressed as follows:

(12) $$\phi \left(d,t\right)=V_{\mathrm{appl}}\left(t\right)=k_{\mathrm{ramp}}t_{\mathrm{ramp}}\;$$

Here *V*_appl_ is the external applied voltage in V; *k*_ramp_ is the ramp rate of voltage source in kVs^−1^; and *t*_ramp_ is the applied time of ramping voltage in s. After obtaining the electric potential in the polymeric material, we can calculate the electric field by the definition of electric field, *F* = −∇*φ*.

#### 4.1.2. Parameters for SCEB Model

The thickness range of polyimide samples used in the simulations was similar to the experiments, namely from 25 to 250 μm. The simulation temperature was the same as that during the breakdown measurements, which was around 303 K. The energy of deep traps was extracted from the TSDC experimental results. The density and energy of deep traps for electrons and holes were assumed to be the same value, which were 6.25 × 10^20^ m^−3^ and 0.83 eV, respectively. The effective injection barriers of electrons and holes were 0.98 eV. The carrier mobilities of electrons and holes controlled by shallow traps in polyimide were 3.67 × 10^−14^ m^2^V^−1^s^−1^ and 1.80 × 10^−14^ m^2^V^−1^s^−1^, respectively, which were obtained by the SPD measurements. The relative permittivity of polyimide used in the simulations was 3.5.

#### 4.1.3. Simulation Results of SCEB Model

In the SCEB model, it is assumed that electrical breakdown occurs when the maximum local electric field *F*_max_ inside the material exceeds a threshold *F*_c_. If the space charge effect is not considered, the internal electric field distributes evenly or is equal everywhere. Consequently, the electric field inside the sample increases linearly with an increase in the application time of a ramp voltage. The breakdown time *t*_b_ is proportional to the sample thickness *d*, namely, *t*_b_ = *F*_c_*d/V*_ramp_. Based on the assumption of no space charge accumulated in the dielectric material, the electrical breakdown strength will not vary with the sample thickness, namely, *F*_b_ = *V*_ramp_*t*_b_*/d = F*_c_. In other words, the breakdown strength is independent of material thickness. Obviously, this ideal situation does not match the experimental results.

**Figure 6:**
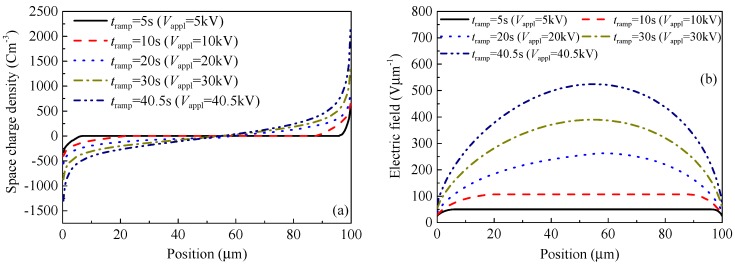
Numerical results of space charge and electric field profiles as a function of position. (**a**) The distribution of space charges (**b**) and electric fields at various times. The ramping rate of the applied voltage is 1 kVs^−1^, the cathode and the anode are located at *x* = 0 and 100 μm, respectively, *t*_ramp_ is the elapsed time of voltage, and *V*_appl_ represents the instantaneous voltage applied to the sample.

**Figure 7:**
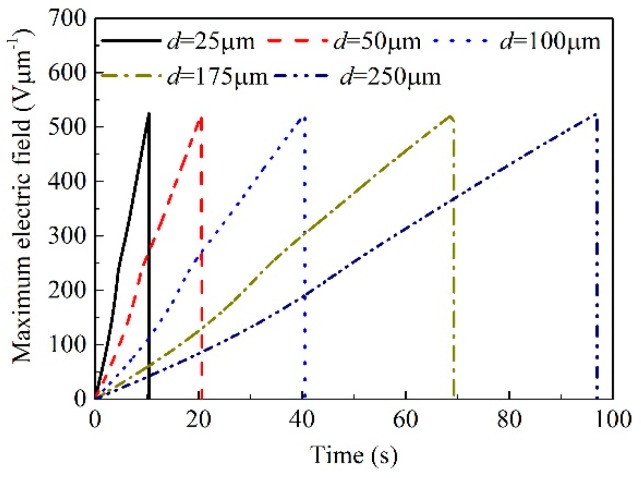
Numerical results of the maximum local electric field as a function of time in polyimide samples with various thicknesses ranging from 25 to 250 μm.

The SCEB model is then used to simulate the DC electrical breakdown process of polyimide under the influence of space charge accumulation and electric field distortion. After charges are injected into the dielectric material from the electrodes, they may be captured by traps formed by carbonyl groups [37,38] or by the energy level difference between pyromellitic dianhydride and 4.4’ diaminodiphenyl ether [39]. Then, space charges are accumulated inside the material, resulting in the distortion of electric fields. Figure 6 shows how the distribution of space charges and electric fields in a polyimide film with a thickness of 100 μm depend on the time *t* elapsed after the application of a ramp voltage *V*_ramp_. It can be seen from Figure 6 that the amount of space charges accumulated in the material increases and the distortion of the electric field becomes serious with the elapsed time after the application of voltage. As shown in Figure 6a, at the initial moment, the applied voltage is very low, so there is little charge injection. The internal electric field is determined by the combination of the applied electric field and the accumulated space charges. At the beginning, the internal electric field is almost determined by the applied voltage, resulting in a uniform distribution, as shown in Figure 6b. As increasing the applied voltage, charges are injected continuously into the material and the space charge accumulation leads to electric field distortion. For example, at *x* = 10 μm nearby the cathode, the space charge density is −148.8 Cm^−3^ at *t* = 150 s, and the space charge density is −408.3 Cm^−3^ at *t*_b_ = 403 s just before the occurrence of electrical breakdown. The electric field distortion factor *α* that is defined as the ratio between the maximum local electric field and the average electric field, *α* = *F*_max_/*F*_appl_, is 1.2 at *t* = 150 s, and 1.3 at *t*_b_ = 403 s. Since the mobility of holes is smaller than that of electrons, more positive charges accumulate near the anode than negative charges near the cathode. According to Poisson’s equation, the derivative of the electric field with respect to position is proportional to the density of accumulated charges. Therefore, the position at which the maximum electric field occurs is biased toward the anode. The maximum local electric field increases with time as shown in Figure 7, and electrical breakdown occurs when the maximum local electric field exceeds 525 Vμm^−1^. The simulation results indicate that the DC electrical breakdown starts from the middle of the material, which is consistent with the observation by pulsed electroacoustic apparatus [20]. The effect of space charge accumulation on electrical breakdown was also observed in poly(vinylidene fluoride-trifluoroethylene-chlorofluoroethylene) terpolymer. It was found that the electrical breakdown field decreases with an increase in the charge injection from electrodes into the material [40].

**Figure 8:**
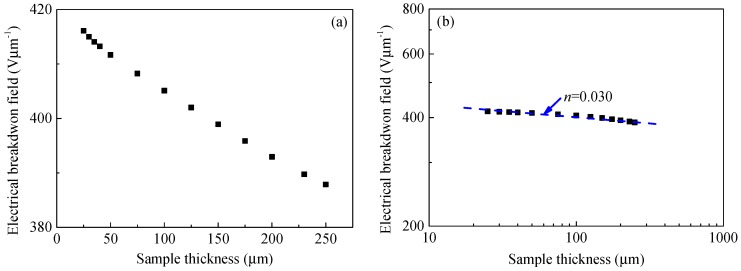
Numerical results of the DC electrical breakdown field as a function of sample thickness of polyimide in the linear coordinates (**a**) and the double logarithmic coordinates (**b**). Symbol ■ represents the simulation results of the space charge modulated electrical breakdown (SCEB) model, while **- - -** is a fitting curve calculated by an inverse power function.

Figure 8 demonstrates the DC electrical breakdown field of polyimide as a function of sample thickness obtained from the calculations by the SCEB model. It is obvious that the DC electrical breakdown field decreases with an increase in sample thickness. As shown in Figure 8a, the DC electrical breakdown field is 416 Vμm^−1^ at the sample thickness of 25 μm, 405 Vμm^−1^ at 100 μm, and 388 Vμm^−1^ at 250 μm. The DC electrical breakdown field at the sample thickness of 25 μm is 28 Vμm^−1^ higher than that at 250 μm. Chen et al. obtained similar results on low-density polyethylene [14]. The DC electrical breakdown field is 419 Vμm^−1^ at the sample thickness of 25 μm, 406 Vμm^−1^ at 100 μm, and 399 Vμm^−1^ at 250 μm. The difference between the DC electrical breakdown field at the sample thicknesses of 25 μm and that at 250 μm is 20 Vμm^−1^ [14].

Figure 8b shows the DC electrical breakdown field as a function of sample thickness in double logarithmic coordinates. The numerical results obtained from the SCEB model show that the breakdown strength *F*_b_ and sample thickness *d* satisfies an inverse power function, *F*_b_ = *kd*^−*n*^, and the inverse power index *n* equals to 0.030. However, the inverse power function curve obtained by the SCEB simulation is far from the experimental results. The inverse power index *n* of SCEB simulation is much lower than that obtained from experiments, which is 0.324 as shown in Figure 4b. Therefore, the CTMD model that considers the displacement of the molecular chain with trapped charges causing an enlargement in free volume is used to further study the relationship between the breakdown strength and sample thickness of polyimide.

### 4.2. Numerical Analysis by Charge Transport and Molecular Displacement Modulated (CTMD) Model

#### 4.2.1. Dynamics of Molecular Displacement under Electric Field

**Figure 9:**
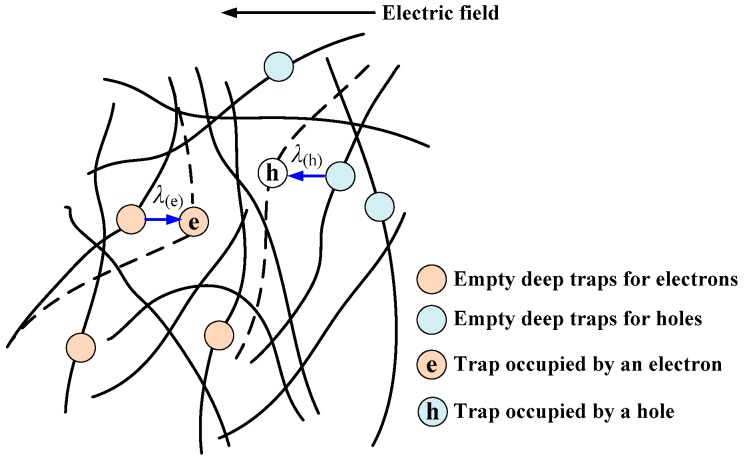
Schematic diagram of the displacement of molecular chains with occupied deep traps under an electric field. The molecular chains with positive and negative trapped charges move toward the cathode and anode, respectively.

The magnitude of molecular displacement depends on temperature, electric field, and mechanical strength. The motion of molecular chains will be accelerated by increasing the temperature, electric field, or mechanical force [23,41]. In the experiment of DC electrical breakdown, the electric field is high enough to facilitate the motion of molecular chains. On the one hand, if a molecular chain has a dipole moment, the chain will rotate under the external electric field. On the other hand, when a molecular chain with deep traps formed by chemical defects captured charge carriers, a Coulomb force due to the external electric field will act on the chain [24,25]. As shown in Figure 9, molecular chains with negative carriers will move toward the anode, while those with positive carriers will move toward the cathode. The displacement of a molecular chain is an integral of time, so it depends on the retention time of charges in trap centers or the actuation duration of the Coulomb force on the molecular chain. Since the retention time of charges in trap centers increases exponentially with an increase in trap energy, the charges reside longer in the deep traps, which means that the Coulomb force acts a longer period on molecular chains with occupied deep traps compared to molecular chains with occupied shallow traps. The displacement of a molecular chain with trapped charges in the dielectric material will enlarge the local free volume. Electrical breakdown occurs when the energy of electrons gained from electric field in the enlarged free volume is greater than the energy of deep traps. It can be concluded that the displacement of a molecular chain with occupied deep traps would determine the electrical breakdown strength of the material. Consequently, we will focus on the molecular chains with deep traps in the following numerical simulations. The velocity equation for the motion of a molecular chain with trapped charges is expressed as [24,26],

(13) $$d\lambda /dt=\mu_{\mathrm{mol}}F-\lambda /\tau_{\mathrm{mol}}$$

where *λ* is the displacement of a molecular chain in m; *μ*_mol_ is the mobility of molecular chains in m^2^V^−1^s^−1^; and *τ*_mol_ is the relaxation time constant of molecular chains in s. The mobility of molecular chains is determined by the shallow trap controlled carrier mobility and the energy of deep traps, namely *μ*_mol_ = *μ*_0_*P*_de_/(*P*_tr_ *+ P*_de_). The relaxation time constant of molecular chains equals the retention time of charges in deep traps, namely *τ*_mol_ = *τ*_0_exp(*E*_T_/*k*_B_*T*).

In order to calculate the displacement of molecular chains, we need to determine electric field distribution in the material firstly. Then, we can obtain the velocity and displacement of the molecular chain by solving Equation (13). Therefore, we used a CTMD to simulate the space charge distribution and molecular chain motion in polyimide.

#### 4.2.2. Simulation Results of CTMD Model

**Figure 10:**
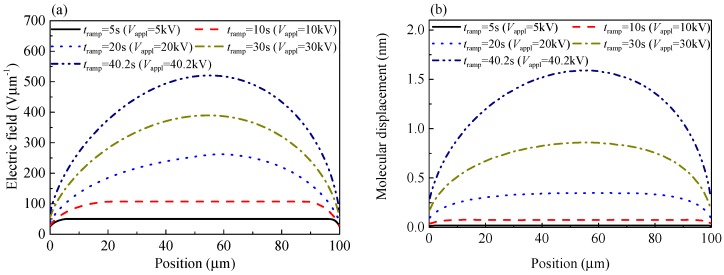
Numerical results of the electric field and molecular chain displacement as a function of position. The distributions of electric field (**a**) and molecular chain displacement (**b**) at various times. The ramping rate of voltage is 1 kVs^−1^, the cathode and the anode are located at *x* = 0 and 100 μm, respectively, *t*_ramp_ is the elapsed time of ramp voltage, and *V*_appl_ represents the instantaneous voltage applied to the sample.

Figure 10 shows how the electric field distributions and molecular chain displacement distributions depend on the time *t*_ramp_ elapsed after the application of a ramp voltage at the sample thickness of 100 μm for example, according to the calculations by the CTMD model. As the applied voltage *V*_appl_ rises, more and more space charges are injected and accumulated inside the sample, which can distort the electric field. As shown in Figure 10a, the electric field has a convex shape with a maximum value in the middle of the sample in the case of homo space charges. The maximum local electric field increases non-linearly with the elapsed time of voltage. For example, the maximum local electric field is 107 Vμm^−1^ at 10 s, 262 Vμm^−1^ at 20 s, and 520 Vμm^−1^ at 40.2 s. Molecular chains with negative trapped charges move toward the anode, while those with positive trapped charges move toward the cathode. When the displacement is not very long or the relaxation time constant of molecular chains is very large, the second term on the right side can be neglected according to the molecular chain displacement Equation (13), and the velocity of the molecular chain with trapped charges is proportional to the electric field. Therefore, the molecular chain displacement curve has the same tendency as the electric field curve as shown in Figure 10b. The maximum of molecular chain displacement *λ*_max_ appears in the middle of the sample, while the displacement value near the electrodes is relatively small. The maximum local molecular displacement is 0.077 nm at 10 s, 0.35 nm at 20 s, and 1.59 nm at 40.2 s.

**Figure 11:**
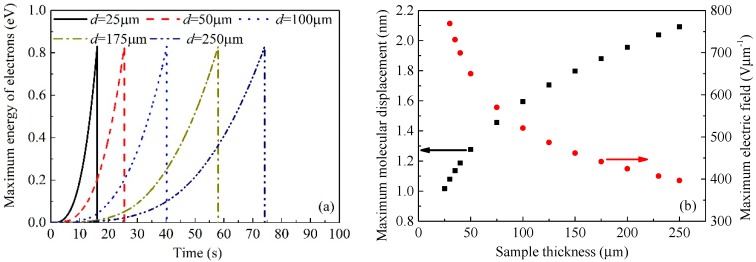
Numerical results of time-dependent maximum energy of electrons gained from the electric field for the samples at various thicknesses (**a**) and sample thickness-dependent maximum local molecular displacement and maximum local electric field at pre-breakdown (**b**).

Figure 11a shows the maximum energy of electrons gained from electric field for the samples at various thicknesses according to the calculations by the CTMD model. The molecular chain with occupied deep traps will move under the electric field, resulting in the extension of free volume inside the sample. In other words, the length of free volume in a dielectric material increases as the molecular chain displacement increases. It is assumed that the length of free volume is very small in the initial state, which can be neglected for polyimide at room temperature which is much lower than its glass transition temperature. Therefore, the length of free volume *λ* is equal to the displacement value of molecular chains under the applied electric field. The molecular chain displacement or the length of free volume increase with the applied voltage increases. The increases in both local electric field and molecular chain displacement lead to the increase in the energy of electrons gained from the electric field in the free volume, as shown in Figure 11a. When the maximum energy of electrons *w*_max_ = (*eFλ*)_max_ exceeds the trap energy level *E*_T_, the local currents would multiply and the temperature rises suddenly, causing electrical breakdown eventually. The influence of molecular chain displacement on electrical breakdown field was also evidenced in poly(vinylidene fluoride-hexafluoropropylene) copolymer [42]. In addition, the simulation results of the CTMD model indicate that the breakdown occurs at the position where the electric field is the highest as shown in Figure 10.

When the molecular displacement is not very large, Equation (13) can be simplified to be d*λ*/d*t* = *μ*_mol_*F*. Replacing *F* by *k*_ramp_*t*/*d* and considering the initial condition *λ*(*t* = 0) = 0, we can obtain *λ* = (*k*_ramp_*μ*_mol_/2*d*)*t*^2^. The molecular displacement or the length of free volume is a function of the reciprocal of sample thickness and the square of time. Generally, the elapsed time after the application of a ramp voltage until the occurrence of breakdown in a dielectric material is proportional to the sample thickness. Since the variation in the square of time is faster than that of sample thickness, the molecular displacement or the length of free volume increases with the increase in sample thickness as shown in Figure 11b. Nevertheless, the derivative of molecular displacement with the respect to sample thickness decreases with the increase in sample thickness. In addition, electrical breakdown occurs when the maximum energy of electrons that is a product of the maximum length of free volume and the maximum electric field exceeds the trap energy level; therefore, the maximum electric field at pre-breakdown decreases with an increase in sample thickness as depicted in Figure 11b.

**Figure 12:**
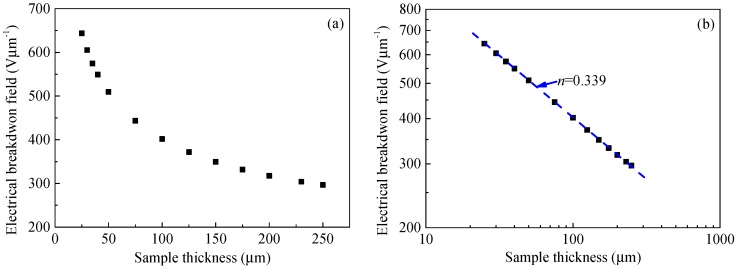
Numerical results of the DC electrical breakdown field as a function of sample thickness of polyimide in linear coordinates (**a**) and in double logarithmic coordinates (**b**). Symbol ■ represents the simulation results of the SCEB model, while **- - -** is a fitting curve calculated by an inverse power function.

Figure 12 demonstrates the DC electrical breakdown field of polyimide as a function of sample thickness obtained from the calculations by CTMD model. As shown in Figure 12a, the DC breakdown field decreases with an increase in sample thickness. The DC electrical breakdown field is 643 Vμm^−1^ at the sample thickness of 25 μm, 402 Vμm^−1^ at 100 μm, and 297 Vμm^−1^ at 250 μm. The DC electrical breakdown field at the sample thickness of 25 μm is 346 Vμm^−1^ higher than that at 250 μm. It can be seen from Figure 12b that there is a linear relationship between the DC breakdown strength and the sample thickness in the log-log coordinates, which can be expressed in the form of an inverse power function *F*_b_ = *kd*^−*n*^. The inverse power index *n* equals 0.339, which is close to the experimental result *n* = 0.324.

### 4.3. Discussion

When the space charge effect is considered as demonstrated by the SCEB model, space charges accumulate inside a dielectric material with an increase in the application time of external voltage and the internal electric field is distorted, which would cause a drop in breakdown strength. Homo space charges accumulate near the interfaces between the dielectric material and its electrodes [18]. Consequently, the electric field near the electrode is weakened, and conversely the electric field at the middle of the material is enhanced. The more homo space charges that accumulate inside the material, the more severely the internal electric field will be distorted or the larger the maximum electric field *F*_max_ will be, which will lead to a decrease in the electrical breakdown field [20]. Generally, when the electrodes and the sample are the same, the accumulation of space charges will increase with an increase in the application time of external voltage, which results in a larger *F*_max_ and a lower breakdown strength. With the sample thickness increasing, the time required for the internal electric field to reach the breakdown threshold becomes longer under the same ramp voltage. This means that the breakdown time of thicker samples is longer than that of thinner samples. The accumulation of space charges inside the thicker sample is greater, and the distortion of the electric field is more severe, which makes the electrical breakdown field of a relatively thicker sample lower than that of the relatively thinner sample [14]. Therefore, the breakdown strength decreases as the thickness increases.

In the CTMD model, molecular chains with occupied deep traps displace under the external electric field, leading to an increase in the length of free volume [24,26]. The voltage applied to the sample increases with a ramping rate; consequently, both the local electric field and the molecular chain displacement or the length of free volume increase with time. Accordingly, electrons will be accelerated to higher energy in the free volume at a relatively longer duration after the application of voltage. When electrons gain enough energy to jump over the potential barrier, the sample will be broken by the accelerated electrons [12,43]. The displacement of a molecular chain with trapped charges under an electric field is the integral of elapsed time after the application of voltage. As the thickness of the sample increases, the time required to reach the breakdown threshold *E*_T_ is prolonged. Consequently, the displacement value of a molecular chain increases with the increase in sample thickness. According to the breakdown criterion *w_max_* > *E*_T_, the increase in the free volume length will cause the decrease in the electric field *F* required for the electron to be accelerated to the energy *E*_T_. Therefore, the electrical breakdown field is inversely related to the sample thickness.

**Figure 13:**
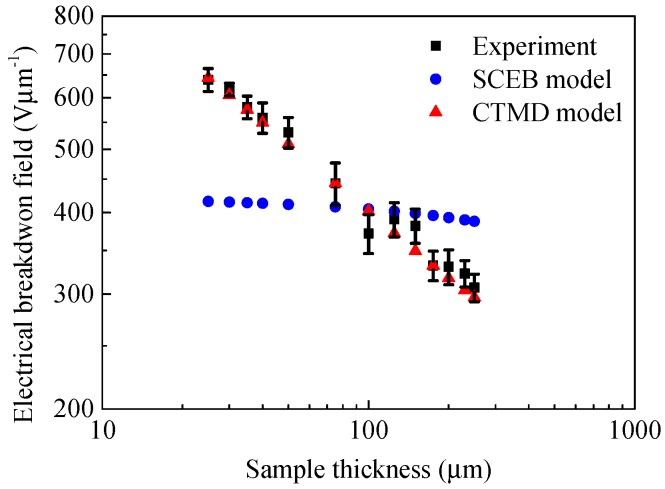
Comparison of experimental results and simulation results obtained by SCEB and charge transport and molecular displacement modulated (CTMD) models on DC electrical breakdown field as a function of sample thickness.

Figure 13 shows a comparison of DC electrical breakdown field as a function of sample thickness calculated by the SCEB model and the CTMD model with the experimental results. It can be seen from the comparison that the simulation results obtained by the CTMD model are in good agreement with the experimental results. However, the breakdown results calculated by the SCEB model deviate significantly from the experimental values. It indicates that DC electrical breakdown is a cumulative process. Therefore, compared with the SCEB breakdown model considering the space charge effect, the CTMD model considering the charge transport and molecular chain displacement can give a better explanation for the DC electrical breakdown characteristics of polyimide.

## 5. Conclusions

The thickness-dependent DC electrical breakdown properties of polyimide were investigated by experiments and simulations of the SCEB model and the CTMD model. The experimental results show that the electrical breakdown field decreases with an increase in sample thickness in the form of an inverse power function, and the inverse power index is *n* = 0.324. The trap properties, carrier mobilities, and complex permittivity were also measured for further investigation into the influencing mechanism of thickness on the electrical breakdown characteristics of polyimide. Then, the SCEB model was used to simulate the electrical breakdown properties of polyimide. Charges are injected into and transport inside the dielectric material under external electric field. During the migration of mobile charges in shallow traps, some of them are captured by deep traps and form space charges. The electric field in the middle of the sample is then enhanced by the accumulation of space charges. When the maximum local electric field exceeds a threshold value, the material is broken. More space charges are accumulated in thicker samples and the electric field is distorted more severely, resulting in the electrical breakdown field decreasing as the sample thickness increases. In the CTMD simulations, both the charge transport and molecular chain displacement dynamics processes were considered. After mobile charges are captured by deep traps formed by chemical defects on molecular chains, the molecular chain with occupied deep traps moves toward the electrode driven by the Coulomb force, which enlarged the free volume. When electrons gain enough energy in the free volume from the electric field, electrical breakdown occurs in polyimide. Moreover, the elapsed time after the application of ramp voltage until the occurrence of electrical breakdown increases with an increase in sample thickness, and the length of free volume is proportional to the square of time; accordingly, the free volume at pre-breakdown is larger at the relatively thicker sample. Since the threshold electron energy is a product of the length of free volume and electric field, the electrical breakdown field decreases with an increase in sample thickness following an inverse power function. The comparison between the experimental results and simulations indicates that the simulation results obtained by the CTMD model are closer to the experiments and the electrical breakdown is modulated by both charge transport and molecular displacement dynamics.

## Figures and Tables

**Table 1:** Parameters extracted from TSDC experimental results by Equation (1).

Peak Temperature (°C)	*E*_A_ (eV)	*B* (Am^−2^)	*τ*_0_ (s)
69	0.60	2.63 × 10^−4^	7.50 × 10^−7^
87	0.65	2.01 × 10^−4^	4.23 × 10^−7^
109	0.70	1.80 × 10^−4^	3.23 × 10^−7^
135.5	0.83	1.60 × 10^−4^	3.09 × 10^−8^
